# An emerging coronavirus causing pneumonia outbreak in Wuhan, China: calling for developing therapeutic and prophylactic strategies

**DOI:** 10.1080/22221751.2020.1723441

**Published:** 2020-01-31

**Authors:** Shibo Jiang, Lanying Du, Zhengli Shi

**Affiliations:** a Key Laboratory of Medical Molecular Virology (MOE/NHC/CAMS), School of Basic Medical Sciences, Fudan University, Shanghai, People’s Republic of China; b Lindsley F. Kimball Research Institute, New York Blood Center, New York, NY, USA; c CAS Key Laboratory of Special Pathogens and Biosafety, Wuhan Institute of Virology, Chinese Academy of Sciences, Wuhan, People’s Republic of China

In December of 2019, an outbreak of pneumonia caused by an unknown aetiology occurred in Wuhan, China and most patients were linked to a single seafood market, which reportedly sold seafood and some live animals, including poultry, bats, marmots and other wild animals, suggesting that the pathogen may be transmitted from an animal to human. The pathogen was soon identified to be a novel coronavirus, 2019-nCoV denoted by WHO [1].

On 19 January 2020, Wuhan Health Commission reported that a total 198 cases in the 25–89-year-old range were confirmed positive for 2019-nCoV, including 25 being discharged and 3 having died. Among the 170 patients under treatment in hospitals, 126, 35, and 9 are in mild, severe, and critical condition, respectively (http://www.thatsmags.com/china/post/30618/new-coronavirus-spreads-to-over-130-in-china-death-toll-rises). In addition, two patients in Thailand, one in Japan, and one in South Korea, were detected positive for 2019-nCoV. They did not visit the specific seafood market, but might have close contact with some pneumonia patients during their trip in Wuhan, raising the concern of limited human-to-human transmission of 2019-nCoV (http://www.thatsmags.com/china/post/30618/new-coronavirus-spreads-to-over-130-in-china-death-toll-rises).

**Figure 1.**
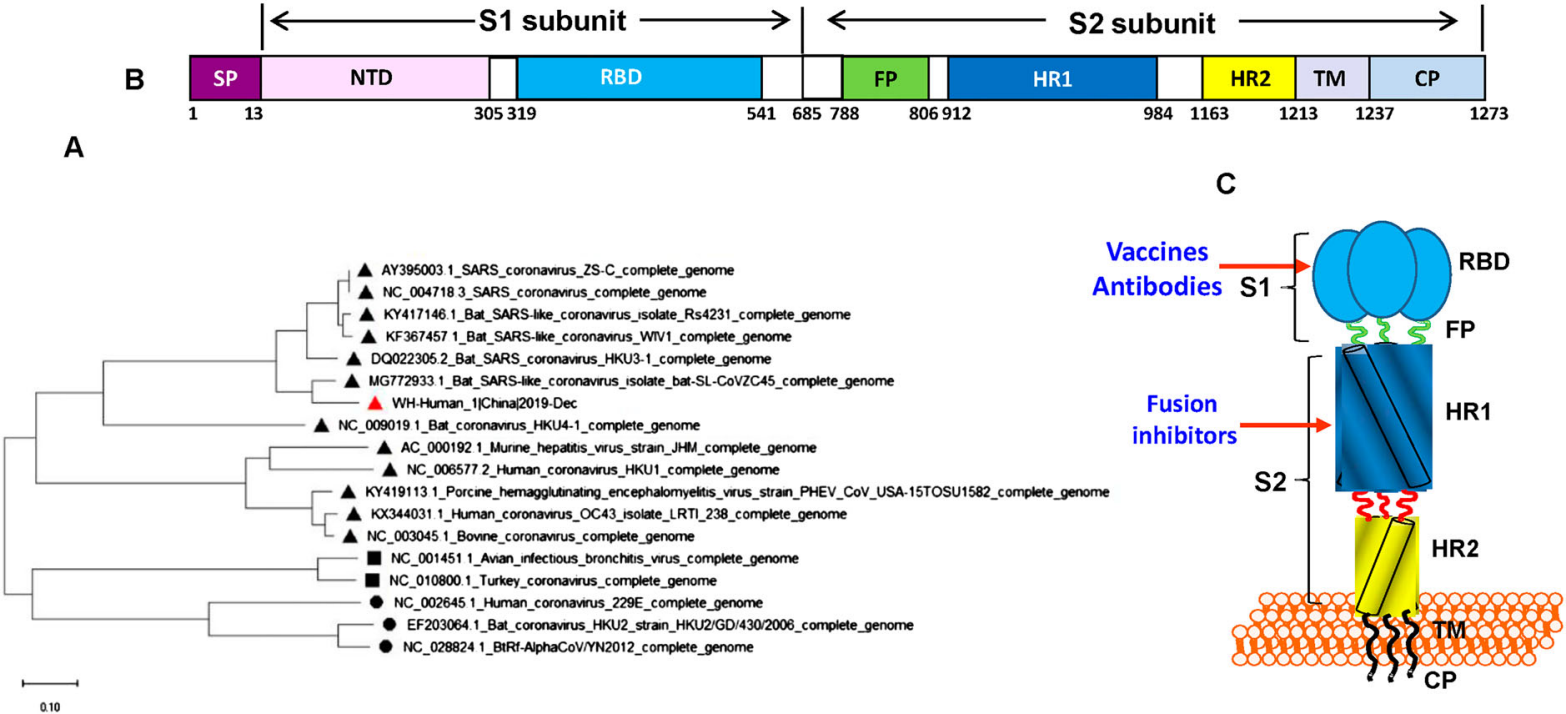
Analysis of the functional domains in 2019-nCoV spike protein and its gene. (A) Phylogenetic analysis of S gene of 2019-nCoV (Wuhan-Hu-1), bat-SL-CoVZXC21, bat-SL-CoVZXC45, SARS-CoV and other coronaviruses using Neighbor-Joining method. (B) The representative scheme of functional domains in S protein of 2019-nCoV. SP, signal peptide; NTD, N-terminal domain; RBD, receptor-binding domain; FP, fusion peptide, HR1, heptad repeat 1; HR2, heptad repeat 2; TM, transmembrane domain; CP, cytoplasmic domain. (C) The target sites in 2019-nCoV S for development of vaccines, antibodies and fusion/entry inhibitors.

Research scientists have released the full genomic sequence of 2019-nCoV, such as Wuhan-Hu-1 (GenBank, accession no. MN908947). The phylogenetic analysis revealed that the gene sequence of 2016-nCoV is 89% identical to that of bat SARS-like coronavirus ZXC21 (bat-SL-CoVZXC21, accession no. MG772934.1) and ZC45 (MG772933.1), and 82% identical to that of SARS-CoV Tor2 (JX163927), suggesting that 2019-nCoV also belongs to betacoronavirus Lineage B, but has closer homology to bat-SL-CoVZC45 and bat-SL-CoVZXC21 than SARS-CoV [2] (Figure 1). Both bat-SL-CoV ZC45 and ZXC21 were found in Chinese horseshoe bats (*Rhinolopus sinicus*) in Zhoushan city of Zhejiang Province, China between 2015 and 2017 [3], which can infect suckling rats and cause disease. Given that there were some bats and live animals in the seafood market, 2019-nCoV may be originated from bats or live animals exposure to the materials contaminated with bat droppings in the seafood market or surrounding area.

The rapid identification of this novel coronavirus is attributed to recent advances in the detection of respiratory virus infection, including reverse transcription PCR (RT-PCR), real-time reverse transcription PCR (rRT-PCR), reverse transcription loop-mediated isothermal amplification (RT-LAMP), and real-time RT-LAMP as well as multiplex nucleic acid amplification and microarray-based assays [4]. These methods are useful for detecting novel coronaviruses not only in humans, but also in animals for identification of animal reservoir or intermediate host of 2019-nCoV. WHO recommended that if there is no clue about the putative pathogen from the pneumonia outbreak, a pan-coronavirus assay should be used for amplification followed by sequencing of the amplicon for characterization and confirmation (https://apps.who.int/iris/bitstream/handle/10665/330374/WHO-2019-nCoV-laboratory-2020.1-eng.pdf). By aligning 2019-nCoV S protein sequence with those of SARS-CoV and several bat-SL-CoVs, we predicted that the cleavage site for generating S1 and S2 subunits is located at R694/S695 (Figure 1). S1 subunit contains two functional domains, the N-terminal domain (NTD) and a receptor-binding domain (RBD), both of which are responsible for the binding of the virion to the receptor on the host cell. They also contain several conformational neutralizing epitopes, serving as a target for developing neutralizing antibodies and vaccines [5]. S2 subunit contains three functional domains, fusion peptide (FP), and heptad repeat (HR) 1 and 2. After binding of RBD in S1 to the receptor, the S2 subunit changes conformation by inserting the FP into the host cell membrane and association between HR1 and HR2 to form six-helical bundle (6-HB), resulting in the fusion between viral and cellular membranes. The viral genetic materials enter into the host cell through the fusion pore for replication in the cell [5]. A peptide derived from the HR2 domain of SARS-CoV S protein (SC-1) can interact with HR1 region in viral S protein to form heterologous 6-HB, resulting in the inhibition of homologous 6-HB formation between HR1 and HR2 domains in viral S protein and thus blocking the viral fusion with the host cell [6]. Since 2019-nCoV S-HR2 sequence is 100% identical to that of SARS-CoV, while there are only a few mutations of non-critical amino acids in S-HR1 region, SC-1 peptide is expected to be also effective against 2019-nCoV infection.

We have recently designed and engineered a pan-CoV fusion inhibitor, EK1 peptide, which could inhibit infection of five human coronaviruses, including SARS-CoV and MERS-CoV, and three bat-SL-CoVs [7]. Intranasal application of EK1 peptide before or after viral challenge, EK1 peptide can protect human DPP4-transgenic mice from MERS-CoV infection, suggesting its potential prophylactic and therapeutic effect against 2019-nCoV infection. Once confirmed, we will develop EK1 peptide as a t prophylactic or therapeutic for intranasal application to prevent or treat infection by 2019-nCoV and other emerging coronaviruses in the future.

The RBDs of SARS-CoV and MERS-CoV contain multiple conformation-dependent neutralizing epitopes that induce more potent neutralizing antibodies and protective efficacy against SARS-CoV and MERS-CoV infections, respectively, than other regions in S protein [5,8,9]. Modification of MERS-CoV S-RBD amino acid residues based on the structure design could improve its protection against MERS-CoV infection [9], suggesting that 2019-nCoV S-RBD or modified S-RBD of other coronavirus may be applied for developing 2019-nCoV vaccines. Of course, the RBD-containing S and S1 of a coronavirus, e.g. 2019-nCoV, can also be used for vaccine development [8].

The recently developed SARS-CoV and MERS-CoV neutralizing monoclonal antibodies (mAbs) and nanobodies with protective efficacy are specific to the S1 subunit of S protein, particularly the RBD [5,8,9–10]. Therefore, the 2019-nCoV S-RBD is anticipated to be a key target for developing 2019-nCoV neutralizing mAbs. The neutralizing mAbs targeting non-RBD regions, including NTD and S2 of SARS-CoV and/or MERS-CoV S could also be identified [5,8,11,12], although their neutralizing potency is generally lower than that of RBD-specific mAbs.

It may take several months or even years for researching and developing neutralizing antibodies against 2019-nCoV infection. One of the rapid approaches is to evaluate the currently available SARS-CoV neutralizing antibodies with cross-neutralizing and protection activity against 2019-nCoV infection. We have shown that SARS-CoV S-RBD-specific neutralizing mAbs and sera could cross-neutralize bat-SL-CoVs, such as bat-SL-CoV-W1V1 and bat-SL-CoV-SHC014 [13], suggesting that they may also cross-neutralize 2019-nCoV. Once identified, these cross-neutralizing antibodies can be promptly developed for urgent prevention and treatment of 2019-nCoV infection.
