# Correction

**DOI:** 10.1080/22221751.2020.1737363

**Published:** 2020-03-05

**Article title:** An emerging coronavirus causing pneumonia outbreak in Wuhan, China: calling for developing therapeutic and prophylactic strategies

**Authors:** Jiang S., Du L., & Shi Z.

**Journal:** *Emerging Microbes & Infections*

**Bibliometrics:** Volume 9, Number 1, pages 275–277

**DOI:** https://doi.org/10.1080/22221751.2020.1723441

When the article was first published online, there were few corrections in Figure 1. This has now been corrected in online.

